# High Altitudes and the Blood Count

**Published:** 1899-10

**Authors:** 


### High Altitudes and the Blood-Count.

In the "Transactions of the American Microscopical Society," Vol. xx, Dr. A. Mansfield Holmes, of Denver, publishes some experiments made at Manitou, Colo., and the summit of Pike's Peak, on the effect of sudden changes of altitude on the number of red blood-corpuscles. Similar observations made hitherto have been as a rule under conditions requiring the lapse of a considerable period of time between the blood-counts at the different altitudes. The immediate effects were therefore less assured, but at Pike's Peak the railroad afforded the means for a very rapid change of altitude of nearly 8,000 feet. He found that the ascent caused at once an increase of 11.5 percent of the red corpuscles, which had increased three hours later, while still on the mountain top, to 14.42 percent. The descent in the evening was accompanied by a decrease of 9.56 percent, though still over 4 percent above what it was before the ascent. It was also found that the blood of a person accustomed to residence at a high altitude was less reduced in red corpuscles than that of one not so acclimatized. Dr. Holmes is inclined to account for the phenomenon, in part at least, by the fact of the diminished amount of oxygen in the rarified air of high altitudes. The organism has to obtain the exygen it demands, and does this by calling into action and circulation many red cells that had been in a quiescent state in the deeper tissues, but he does not attempt to describe how far this or other influences may be responsible. The study has not, as far as we are aware, been before made in this country with the special conditions of rapid change insured by Dr. Holmes, and it is therefore of some interest.

